# Proteomic profiling and biomarker discovery for predicting the response to PD-1 inhibitor immunotherapy in gastric cancer patients

**DOI:** 10.3389/fphar.2024.1349459

**Published:** 2024-05-31

**Authors:** Jiangang Sun, Xiaojing Li, Qian Wang, Peng Chen, Longfei Zhao, Yongshun Gao

**Affiliations:** ^1^ Department of Gastrointestinal Surgery, The First Affiliated Hospital of Zhengzhou University, Zhengzhou, Henan, China; ^2^ Department of Pharmacy, The First Affiliated Hospital of Zhengzhou University, Zhengzhou, Henan, China; ^3^ School of Biomedical Engineering, Shanghai Jiao Tong University, Shanghai, China

**Keywords:** gastric cancer, immune checkpoint inhibitor, proteomics, machine learning, biomarkers

## Abstract

**Background:** Immune checkpoint inhibitors (ICIs) have revolutionized cancer treatment; however, a significant proportion of gastric cancer (GC) patients do not respond to this therapy. Consequently, there is an urgent need to elucidate the mechanisms underlying resistance to ICIs and identify robust biomarkers capable of predicting the response to ICIs at treatment initiation.

**Methods:** In this study, we collected GC tissues from 28 patients prior to the administration of anti-programmed death 1 (PD-1) immunotherapy and conducted protein quantification using high-resolution mass spectrometry (MS). Subsequently, we analyzed differences in protein expression, pathways, and the tumor microenvironment (TME) between responders and non-responders. Furthermore, we explored the potential of these differences as predictive indicators. Finally, using machine learning algorithms, we screened for biomarkers and constructed a predictive model.

**Results:** Our proteomics-based analysis revealed that low activity in the complement and coagulation cascades pathway (CCCP) and a high abundance of activated CD8 T cells are positive signals corresponding to ICIs. By using machine learning, we successfully identified a set of 10 protein biomarkers, and the constructed model demonstrated excellent performance in predicting the response in an independent validation set (N = 14; area under the curve [AUC] = 0.959).

**Conclusion:** In summary, our proteomic analyses unveiled unique potential biomarkers for predicting the response to PD-1 inhibitor immunotherapy in GC patients, which may provide the impetus for precision immunotherapy.

## 1 Introduction

Unprecedented advances have been made in cancer treatment with the use of immune checkpoint inhibitors (ICIs). However, the response to ICIs is limited to a subset of patients (Morad et al., 2021). Different studies on ICI treatment revealed a highly variable objective response rate, ranging from 10% to 23% in gastric cancer (GC) patients (Kang et al., 2017; Shitara et al., 2018; Huang et al., 2019). Therefore, it is urgent to identify the mechanism of resistance to ICI treatment and discover reliable biomarkers capable of predicting the treatment response at the onset of therapy.

Various factors play a crucial role in influencing the response to ICIs. Among these, the expression, landscape, and composition of neoantigens within tumors emerge as robust indicators of the response (McGranahan et al., 2016; Morad et al., 2021). Additionally, oncogenic signaling, metabolic pathways, and their associated mutations have been conclusively demonstrated to drive immunogenic responses across diverse cancer types. Recent studies highlight the potential involvement of extracellular vesicles, specifically the exosome subset, in tumor immunity and resistance to ICIs (Chen et al., 2018; Poggio et al., 2019). Growing evidence suggests that the contribution of the tumor microenvironment (TME), encompassing stromal cells and immune cells, governs immune evasion and resistance to ICIs (Morad et al., 2021).

Biomarkers predictive of the ICI response are under investigation. Many biomarkers, including tumor mutation burden, programmed cell death 1 ligand 1 (PD-L1) expression, microsatellite instability, and Epstein–Barr virus infection status, identify susceptibility to PD-1/PD-L1 inhibitors (Kim et al., 2018; McGrail et al., 2021). However, the results of several clinical trials using these biomarkers at an individual level are not consistent; some are even contradictory (Ji et al., 2019; Kim et al., 2019; Di Bartolomeo et al., 2020). Therefore, to date, no single biomarker is available for adequate patient stratification (not only in GC) due to the complexity of the immune response to cancer.

Machine learning has exhibited significant potential in predicting responses to ICIs, especially when applied to omics data (Polano et al., 2019; Lu et al., 2020; Sung and Cheong, 2022). Mass spectrometry (MS)-based proteomics techniques provide accurate, specific, and high-throughput quantification capabilities. Moreover, the proteomic layer more precisely reflects cellular function. Despite these advantages, limited research has explored the application of MS-based proteomics techniques to identify predictive biomarkers for ICI response (Longuespée et al., 2023). To the best of our knowledge, no study has yet integrated machine learning and proteomics in the context of GC for this purpose.

In this study, clinical GC tissue samples were collected from a cohort of 28 GC patients before initiating treatment with camrelizumab. Through proteomic data analysis, we aimed to elucidate the underlying mechanisms that may influence patient response, including relevant signaling pathways and the TME. Additionally, by employing machine learning techniques, we successfully identified a panel of biomarkers and developed a robust prediction model, which was subsequently validated on an independent dataset. We are confident that the findings from this study will contribute to the advancement of personalized cancer treatment and ultimately lead to improvements in patients’ quality of life.

## 2 Results

### 2.1 Clinical–pathological features and sample processing of the GC patients

Between June 2019 and May 2021, a total of 28 GC patients were enrolled in this study. Following the completion of immunotherapy, 17 patients exhibited a favorable response to PD-1 inhibitors, while the remaining 11 patients did not manifest a similar response. As shown in Table 1, the median age of the enrolled patients was 59 years (range: 37–78 years), with 64.2% being men. Tumors had invaded the outer lining of the stomach (T4) in 85.7% of cases, and metastasis to other parts of the body (M1) was observed in 78.5% of cases. No significant association was found between the response to immunotherapy and clinical–pathological characteristics.

**TABLE 1:** Baseline characteristics of gastric cancer (GC) patients.

	Progressors (*n* = 11)	Responders (*n* = 17)	*p*-value
Sex (%)			
Female	4 (36.4)	6 (35.3)	1
Male	7 (63.6)	11 (64.7)	
Age [mean (SD)]	57.91 (11.72)	59.00 (8.69)	0.779
Degree of differentiation (%)			
Moderate–poorly differentiated	4 (36.4)	7 (41.2)	0.497
Moderately differentiated	1 (9.1)	4 (23.5)	
Poorly differentiated	6 (54.5)	6 (35.3)	
Lauren’s criteria (%)			
Diffuse	8 (72.7)	7 (41.2)	0.206
Intestinal	1 (9.1)	6 (35.3)	
Mix	2 (18.2)	4 (23.5)	
T (%)			
1	0 (0.0)	1 (5.9)	0.361
2	0 (0.0)	3 (17.6)	
4	11 (100.0)	13 (76.5)	
N (%)			
0	1 (9.1)	7 (41.2)	0.215
1	3 (27.3)	5 (29.4)	
2	3 (27.3)	1 (5.9)	
3	4 (36.4)	4 (23.5)	
M (%)			
0	1 (9.1)	5 (29.4)	0.355
1	10 (90.9)	12 70.6)	

**FIGURE 1:**
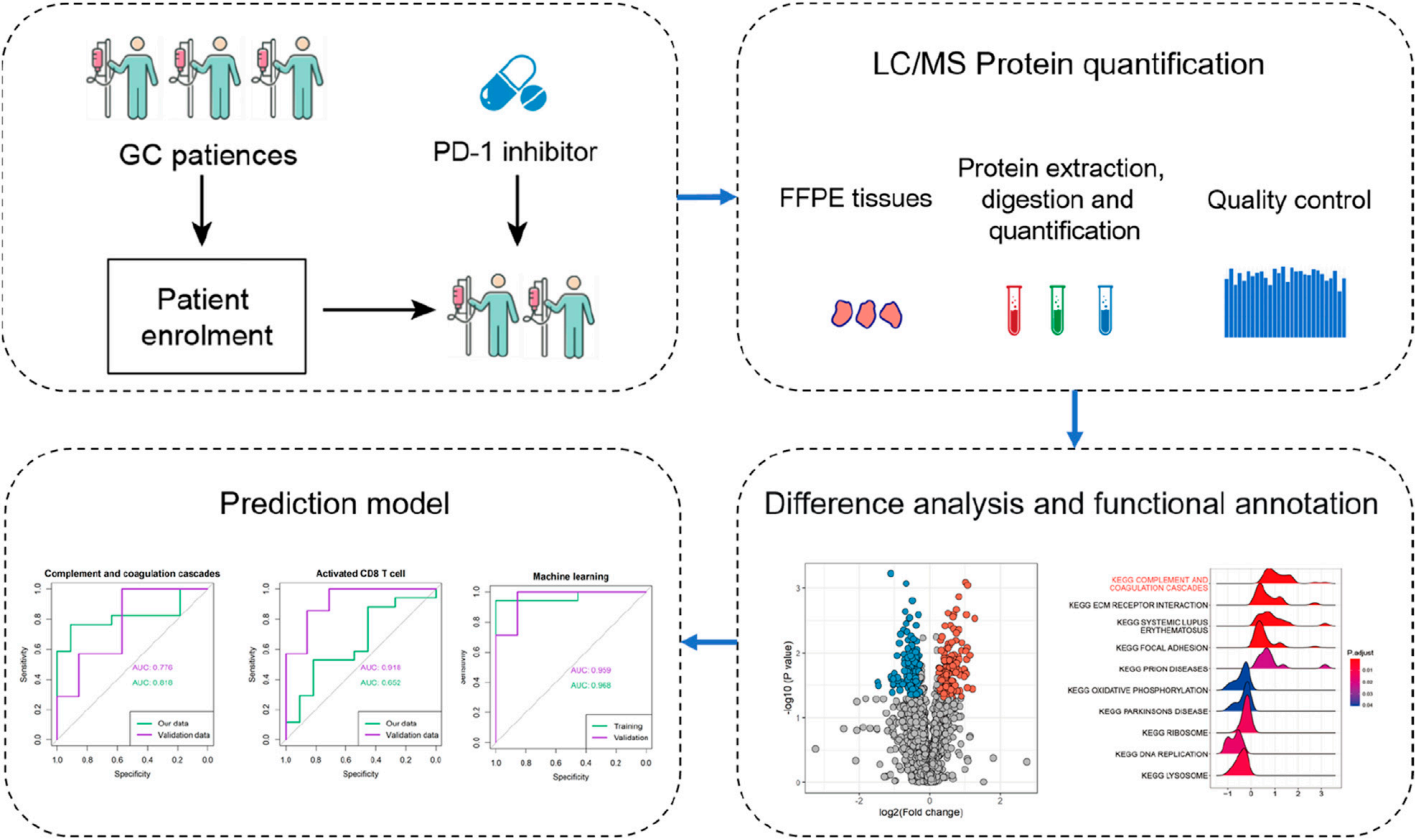
Schematic representation of the experimental design in this study.

Archival pre-treatment tissue specimens were available for all patients. The protein was extracted from formalin-fixed, paraffin-embedded (FFPE) tissues and subjected to quantitative analysis using MS. Stringent quality control measures were implemented to ensure the reliability of the data (see Methods for details, Supplementary Figure S1). Details of the comprehensive study design are given in Figure 1.

### 2.2 Differences in protein expression between responders and non-responders to immunotherapy

**FIGURE 2:**
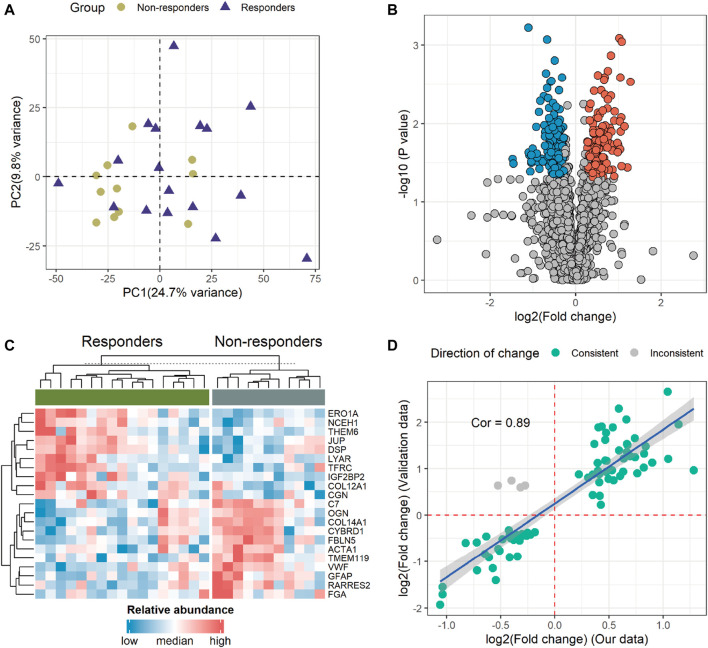
Identification and profiling of differentially expressed proteins (DEPs). **(A)** The principal component analysis (PCA) illustrates the overall variance between the two groups. **(B)** Volcano plot demonstrating 320 DEPs. The red and blue dots indicate proteins with high and low expressions in non-responders, respectively. **(C)** Heatmap displaying the 21 proteins that significantly differed (*p* < 0.05; fold change > 2 or < 1/2) between the two groups. **(D)** Correlation analysis of DEPs between our data and validation data. The green and gray dots denote consistent and opposing directions of change, respectively. Cor, correlation coefficient.

To gain an insight into the proteomic profile differences between responder and non-responder groups, we conducted differential expression analysis. First, our principal component analysis (PCA) revealed no significant outliers and minor intergroup variance, suggesting limited distinction among the sample sets (Figure 2A). Subsequently, by employing Student’s *t*-test with a statistical threshold (*p*-value < 0.05; fold change > 1.2 or < 1/1.2), we identified 320 differentially expressed proteins (DEPs), consisting of 139 upregulated and 181 downregulated proteins in non-responders (Figure 2B; Supplementary Table S5). The most significantly altered DEPs included ERO1A, NCEH1, THEM6, JUP, DSP, LYAR, TFRC, IGF2BP2, COL12A1, CGN, C7, OGN, COL14A1, CYBRD1, FBLN5, ACTA1, TMEM119, VWF, GFAP, RARRES2, and FGA. These protein expression profiles across diverse patients are illustrated in Figure 2C. Notably, in non-responders, genes exhibiting higher expression levels tended to correlate with poorer prognoses in GC patients (Supplementary Figure S3). Importantly, upon incorporating validation data from an external experiment following a similar methodology, we observed a strong correlation and concordance in our findings (Figure 2D).

### 2.3 Functional enrichment analysis with DEPs

**FIGURE 3:**
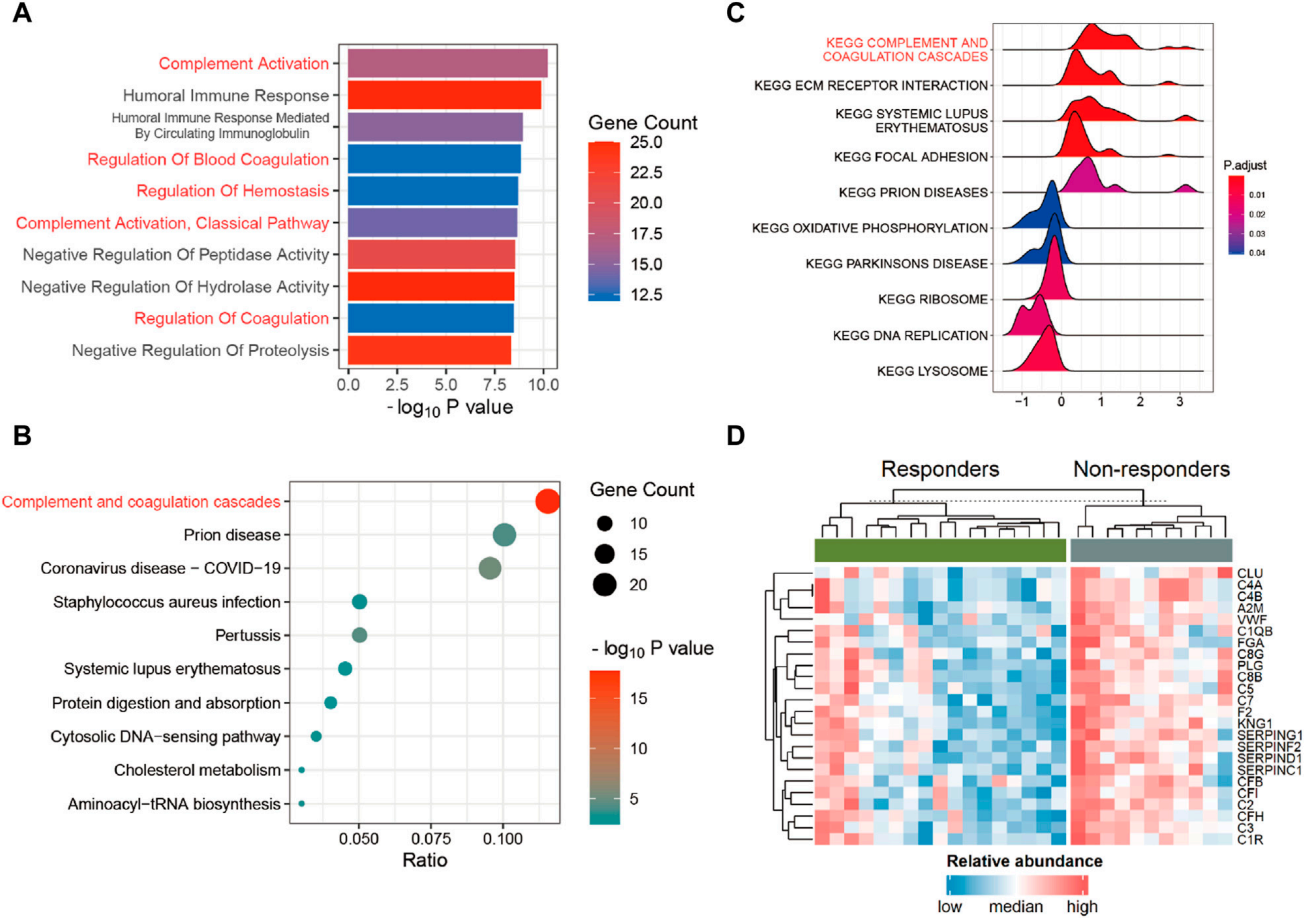
Functional enrichment analysis. **(A)** Gene Ontology (GO) enrichment analysis in DEPs. **(B)** Kyoto Encyclopedia of Genes and Genomes (KEGG) enrichment analysis in DEPs. **(C)** Gene Set Enrichment Analysis (GSEA) in all proteins. **(D)** Heatmap of DEPs within the complement and coagulation cascades pathway (CCCP) between the responders and non-responders.

To gain a deeper understanding of the pathways associated with these DEPs, we conducted enrichment analyses using Gene Ontology (GO), Kyoto Encyclopedia of Genes and Genomes (KEGG), and Gene Set Enrichment Analysis (GSEA). The outcomes of the GO enrichment analysis unveiled that the DEPs are primarily implicated in processes such as “complement activation,” “humoral immune response,” “regulation of blood coagulation,” “collagen-containing extracellular matrix,” and “blood microparticle” pathways (Figure 3A; Supplementary Table S6). Corresponding with the findings from both KEGG (Figure 3B; Supplementary Table S7) and GSEA (Figure 3C; Supplementary Table S8), the complement and coagulation cascades pathway (CCCP) emerged as notably significant. As shown in Figure 3D, nearly all genes within the CCCP exhibited upregulation in non-responders. A detailed exploration of the inter-regulatory relationships among proteins within the CCCP is given in Supplementary Figure S4.

### 2.4 Impact of the TME on the response to immunotherapy

**FIGURE 4:**
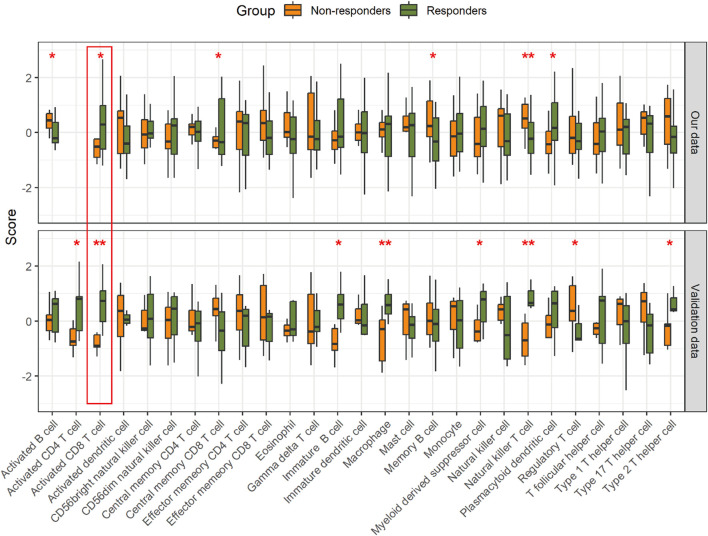
Comparison of the relative abundance of immune cell infiltration in the tumor. *p < 0.05 and **p < 0.01.

The hallmarks of the response and resistance to the immune checkpoint blockade were amplified by a cross-talk between tumor cells and stromal and immune cells within the TME (Morad et al., 2021). Therefore, we observed an association between the ICI response and the TME. To investigate this relationship, we employed single-sample Gene Set Enrichment Analysis (ssGSEA) to assess the differential infiltration levels of various immune cell types (He et al., 2018). We observed significant differences in the abundance of certain tumor-infiltrating immune cells, including activated B cell, activated CD8 T cell, central memory CD8 T cell, memory B cell, natural killer T cell, and plasmacytoid dendritic cell, between the two groups. Notably, activated CD8 T cells showed consistency in the validation data (Figure 4; Supplementary Table S9).

### 2.5 Prediction of patient response to immunotherapy

**FIGURE 5:**
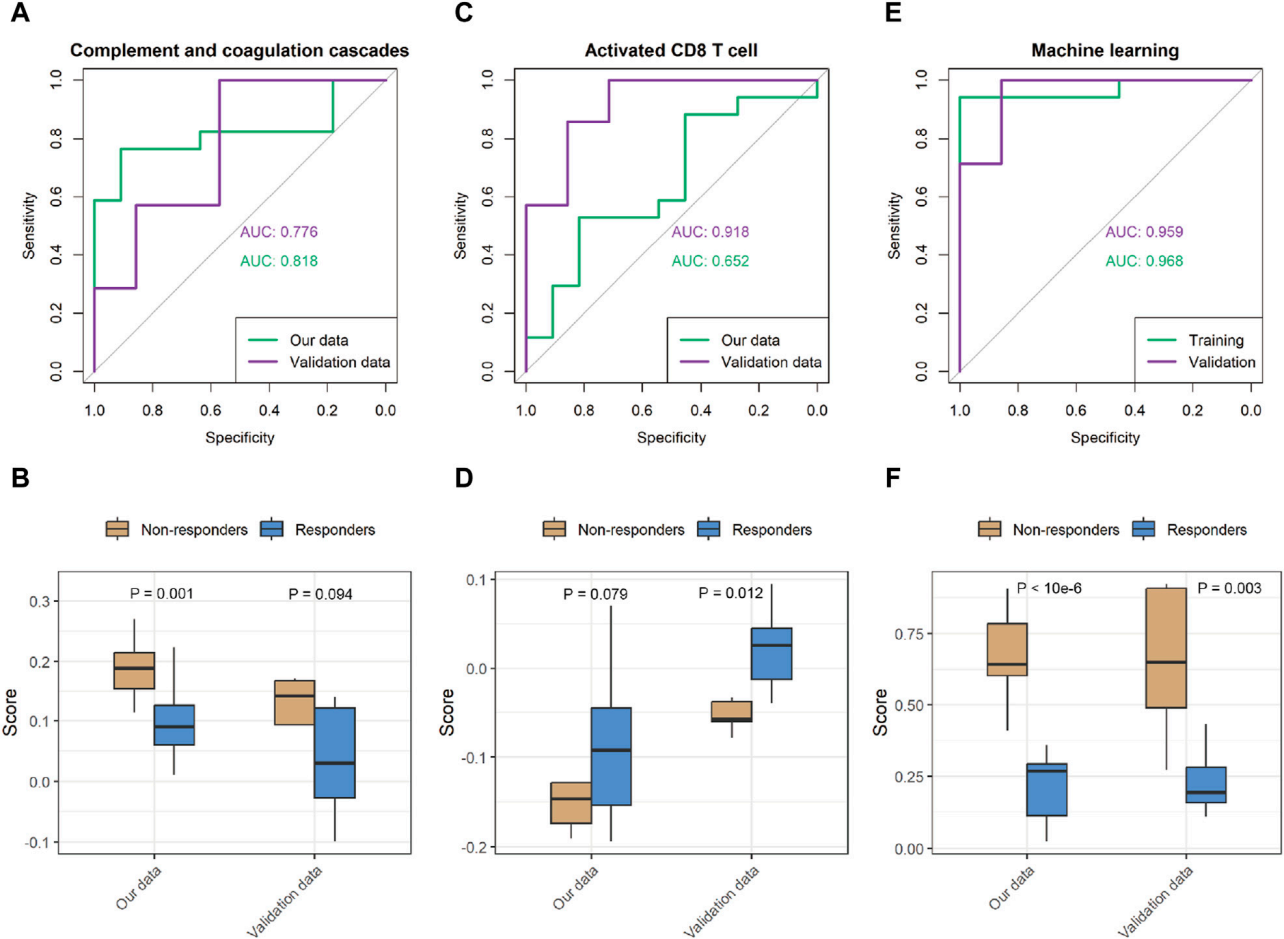
Performance of the prediction model in our data and validation data. **(A,B)** Representative receiver operating characteristic (ROC) curve with the CCCP score as the predictor, and comparison of the predicted score. **(C,D)** ROC curve with the score of the activated CD8 T-cell signature as the predictor, and comparison of the predicted scores. **(E,F)** ROC curve of the machine learning prediction score, and comparison of the predicted scores.

Although some genes with significant changes also exhibit discriminative patterns (Supplementary Figure S5), individual indicators tend to lack robustness. To improve the biological comprehensibility of our predictive model, we evaluated the potential of utilizing pathway enrichment scores and levels of immune cell infiltration as predictors of patient response to immunotherapy. To provide clarity, we plotted the receiver operating characteristic (ROC) curves for the CCCP and activated CD8 T-cell infiltration. With the CCCP as the predictor, the AUC values for our data and validation data were 0.82 and 0.78, respectively (Figures 5A, B). With the level of activated CD8 T-cell infiltration as the predictor, the AUC values for our data and validation data were 0.65 and 0.92, respectively (Figures 5C, D).

While both predictors show some potential for the response to immunotherapy, we further sought to harness the power of machine learning algorithms to achieve superior predictive performance. Specifically, we implemented support vector machines (SVMs) and optimized the performance of our predictive model (Supplementary Table S10). Table 2 shows the performance achieved by our predictive model on the different datasets. The ROC curves show that our model achieved AUC values of 0.97 and 0.96 in our data and the validation data, respectively, indicating excellent prediction accuracy and robustness across datasets (Figures 5E, F).

**TABLE 2:** Performance metrics for the predictive model.

	Accuracy	Precision	Recall	F1 score
Our data	0.893	0.900	0.818	0.857
Validation data	0.857	1.000	0.714	0.833

## 3 Discussion and conclusion

Immunotherapy has emerged as a promising approach for treating various malignancies, leveraging the patient’s immune system to target and eliminate cancer cells. However, not all patients respond uniformly to immunotherapy, and predicting individual treatment responses remains a challenge. Therefore, there is a need to identify more convenient and reliable biomarkers for predicting the benefits of ICIs in clinical practice.

We have pinpointed several differentially expressed proteins with high credibility between responders and non-responders to immunotherapy (Figure 2C). Notably, the expression of the ERO1A protein was significantly elevated in responders. *In vivo* studies demonstrated that ERO1A overexpression promoted tumor growth by suppressing antitumor immunity, acting in collaboration with protein disulfide isomerase (Kukita et al., 2015). The inhibition of ERO1A in tumors might have a synergetic antitumor effect on the immune checkpoint blockade by turning the tumor immunogenic and removing immune-suppressive signals, thereby restoring the antitumor capacity of the T cells in tumor hosts (Liu et al., 2023). Therefore, individuals with higher ERO1A levels may exhibit increased responsiveness to ICIs. ERO1A not only serves as a predictive biomarker but also emerges as a promising therapeutic target for cancer treatment (Johnson et al., 2020). Furthermore, in melanomas, the systemic levels of VWF antigen were measured, confirming VWF as a biomarker of ICI response and overall prognosis (Stadler et al., 2023). Our results also underscore the robust predictive power of VWF for ICIs (Supplementary Figure S5). Similarly, the high levels of GFAP in non-responders also deserve attention. Our findings also validate GFAP as a biomarker for both ICI response and prognosis. These robust results not only provide new evidence for existing studies but also support the value of the other new markersidentified. These molecules hold promise as potential predictive factors for immunotherapy or as novel therapeutic targets.

Our results show that DEPs are enriched in the CCCP. Previously, many studies of preclinical models of lung, colon, and liver cancers have indicated that inflammatory mediators derived from the complement system, such as C5a, together with PD-1 blockade, markedly reduce tumor growth and metastasis, leading to prolonged survival by enhancing antitumor CD8 T-cell responses (Wang et al., 2016; Ajona et al., 2017; Zha et al., 2017). Moreover, cancer cells can exploit the CCCP to shape the TME, thus impacting the efficacy of ICIs (Afshar-Kharghan, 2017; Ruf and Graf, 2020). Another study directly proved that CCCP risk score is an independent biomarker that predicts the efficacy of ICIs in metastatic urothelial cancer patients (Gong et al., 2023). Our results suggest that the CCCP may serve as a potential biomarker in GC.

The TME influences the response to ICIs. ICIs take advantage of immune cell infiltration in the tumor to reinvigorate an efficacious antitumoral immune response (Petitprez et al., 2020; Zhang and Zhang, 2020). By exploring the TME, we found that natural killer T cells, activated CD8 T cells, and memory B cells have an impact on the response to immunotherapy in GC patients. However, only activated CD8 T cells showed consistency between our data and the validation data. It is clear that the presence of infiltrating CD8 T cells in combination with increased PD-L1 expression/amplification is positively associated with the therapeutic efficacy of the PD-1 blockade (Raskov et al., 2021; Chen et al., 2022). In a study of various cancers, the abundance of CD8 T cells within a tumor was found to be the best predictive factor for the response to anti-PD-1/PD-L1 therapy (Lee and Ruppin, 2019). As expected, when protein data were used to infer the extent of CD8 T-cell infiltration, excellent response prediction ability was also shown in GC. However, different types of natural killer T cells, primarily type I and type II, may play completely opposite roles in tumor progression (Terabe et al., 2000; Terabe et al., 2003; Pilones et al., 2012; Robertson et al., 2014; Altman et al., 2015; McEwen-Smith et al., 2015; Nair and Dhodapkar, 2017). Therefore, interpreting the degree of natural killer T-cell infiltration in the context of immunotherapy responses necessitates a more in-depth investigation.

While some molecules and pathways have demonstrated the ability to predict the response to ICIs, a comprehensive and diverse panel of markers providing comparable prognostic accuracy is desirable for clinical applications. Leveraging the capabilities of machine learning, we developed a prediction model utilizing the expression of a specific set of 10 proteins, namely, COL15A1, SAMHD1, DHX15, PTDSS1, CFI, ORM2, VWF, APOA1, EMC2, and COL6A2 (Supplementary Figure S6). The model exhibited robust predictive performance (Figure 5E). Upon validation set assessment, a clear differentiation between responders and non-responders was observed (*p* = 0.003).

While we have demonstrated consistency in the variation between our data and the validation cohort data (Figure 2D), we recognize that the limitations due to the small sample size and the constraints of proteomics technology are significant. Specifically, the limited number of analyzable and overlapping proteins identified across different experimental projects resulted in discrepancies when performing downstream analysis using the DEPs from each dataset (Figure 4). Furthermore, there is a scarcity of available proteomic datasets with the same experimental goals as ours, which restricts the ability to further validate the robustness of our model. Finally, despite the model presenting high sensitivity in predicting immunotherapy efficacy, its application may be limited across diverse cancer types. Tumor heterogeneity and tissue specificity are presumed to be the main reasons. Addressing these issues would require larger sample sizes and more independent datasets. In the future, we will collect many GC samples before immunotherapy to determine the robustness of the model for consequent clinical practice.

Overall, our study revealed that proteomics data and the machine learning method play a critical role in identifying predictive biomarkers that can aid in stratifying patients for immunotherapy. We acknowledge the need for more extensive and in-depth validation studies to translate these findings into clinical applications.

## 4 Materials and methods

### 4.1 Clinical trial protocol

This study was a retrospective study involving 28 enrolled patients with advanced GC between June 2019 and May 2021 at the First Affiliated Hospital of Zhengzhou University, Zhengzhou, Henan, China. The study obtained the ethical approval of our hospital with number 2020-KY-386. All cases were reexamined independently by senior pathologists, and histological diagnosis was performed based on the WHO classification of central nervous system tumors. The clinical data, including age, sex, treatment, body mass index, and pathology results, were obtained from the medical records of the enrolled patients. All patients and their families provided informed consent. The clinical information is shown in Supplementary Table S1. Patients use camrelizumab (AiRuiKa™), a PD-1 inhibitor being developed by Jiangsu Hengrui Medicine Co., Ltd. (Markham and Keam, 2019), according to the protocol. Responders were defined as patients with a RECIST complete response (CR) or partial response (PR), while non-responders were defined as those with progressive disease (PD) or stable disease (SD).

### 4.2 Tumor sample collection

Tumor tissues were obtained any time before initiation of study treatment. The freshly acquired tissues were first rinsed with physiological saline and subsequently fixed in a 10% formalin solution, followed by embedding in paraffin wax.

### 4.3 MS-based protein quantification

After protein extraction, trypsin digestion, MS analysis, and database search, we obtained the raw data of proteomics. Supplementary Information provides more detailed processing information (Supplementary Method).

### 4.4 Quality control and missing value imputation

Following the database search using MaxQuant, 5,107 proteins were identified, of which 4,061 were quantified (Supplementary Table S2). On average, 3,061 proteins per sample were quantified (Supplementary Figure S1A; Supplementary Table S3). Subcellular distribution analysis conducted through the Hum-mPLoc 3.0 database (Zhou et al., 2017) revealed that the majority of identified proteins were cytoplasmic, followed by nuclear and plasma membrane proteins. This distribution aligns with existing references and likely reflects the actual distribution within the tissue, without any subcellular bias (Supplementary Figure S1B). To control the variation among samples, 28 samples were assessed using Pearson’s correlation. The results displayed high correlations among samples with an average Pearson’s correlation coefficient of 0.827 (Supplementary Figure S1C). To identify samples with abnormal protein abundance distributions, the log2-transformed protein intensities were analyzed. The violin plot demonstrates that each sample exhibited a similar distribution when log2-transformed (Supplementary Figure S1D). Quantitative proteomics experiments based on MS frequently generate data with missing values, which can profoundly affect downstream analyses. To address this issue, we evaluated several methods using NAguideR (Kang et al., 2017) and selected the “SeqKNN” method for imputing missing values (Supplementary Figure S2). This imputation process resulted in a dataset consisting of 2,884 proteins for further downstream analysis across all samples (Supplementary Table S4).

### 4.5 Validation data processing

The validation cohort (https://www.iprox.cn//page/subproject.html?id=IPX0004819001) is a group of GC patients treated with anti-PD1 therapy, including seven responder cases and seven non-responder cases (Shi et al., 2023). The sample is also FFPE, and the proteins were quantified using an MS-based label-free method. To ensure data consistency, we downloaded the raw data and re-performed database search and quantification using MaxQuant with the same parameters. After database searching, 4,263 proteins were quantified. After missing value imputation, 2,472 proteins were used for further downstream analysis.

### 4.6 Statistical analysis

All statistical analyses were performed using R software. The code for proteomics-based analysis is available at https://github.com/longfei8533/Predicting-Response-to-PD-1-Inhibitor. The relationships between the treatment response and clinical–pathological features were evaluated using Wilcoxon’s test for continuous variables and Fisher’s exact test for categorical variables. Pearson’s correlation coefficient was used in the correlation analysis. We used two-sided Student’s *t*-test to evaluate the difference in the log2-transformed protein intensity between groups. The R “clusterProfiler” package (Cell, 2023) was used for GO and KEGG functional enrichment analyses and GSEA.

### 4.7 Immune cell abundance inference

We selected immune-related signatures representing 28 different types of immune cells from the study by Charoentong et al. (2017). To estimate the relative abundance of immune cell infiltration in the tumor based on protein intensity, we utilized the Gene Set Variation Analysis (GSVA) package (Hänzelmann et al., 2013) with the ssGSEA method (Barbie et al., 2009). To compare the differences between responders and non-responders, we conducted unpaired one-sided Student’s *t*-tests.

### 4.8 Construction of prediction models

For the CCCP predictor, we selected the “KEGG_COMPLEMENT_AND_COAGULATION_CASCADES” gene set from MSigDB (Liberzon et al., 2011). For the activated CD8 T-cell predictor, the signature gene set from the study by Charoentong was used. Both prediction methods through the utilization of ssGSEA were used to infer the relative activity of pathway.

Considering the small-sample size issue, we utilized SVM as a machine learning approach from the “e1071” package to predict the patient response to medication. Initially, we aligned our dataset with the validation set by selecting overlapping analyzable proteins and then normalized the data using these proteins. Subsequently, important features were chosen using Boruta (Kursa and Rudnicki, 2010), resulting in the retention of 10 proteins as features. We conducted model training after adjusting the SVM parameters; please refer to the Supplementary Material for the specific parameter values. The classifiers developed from the training cohort were then applied to the validation cohort, and the AUC of each classifier was calculated.

## Data Availability

The datasets presented in this study can be found in online repositories. The names of the repository/repositories and accession number(s) can be found at: [iProx database (https://www.iprox.cn)/IPX0008285000].
