# Supplementary material for: Proteomic profiling and biomarker discovery for predicting the response to PD-1 inhibitor immunotherapy in gastric cancer patients
Source: Front Pharmacol. 2024 May 31;15:1349459. doi: 10.3389/fphar.2024.1349459 (PMC11176556; doi:10.3389/fphar.2024.1349459)
Supplement: Supplementary file 1 [file DataSheet1.docx]

**Proteomic Profiling and Biomarker Discovery for Predicting Response to PD-1 Inhibitor Immunotherapy in Gastric Cancer Patients**

**Supplementary Figure**


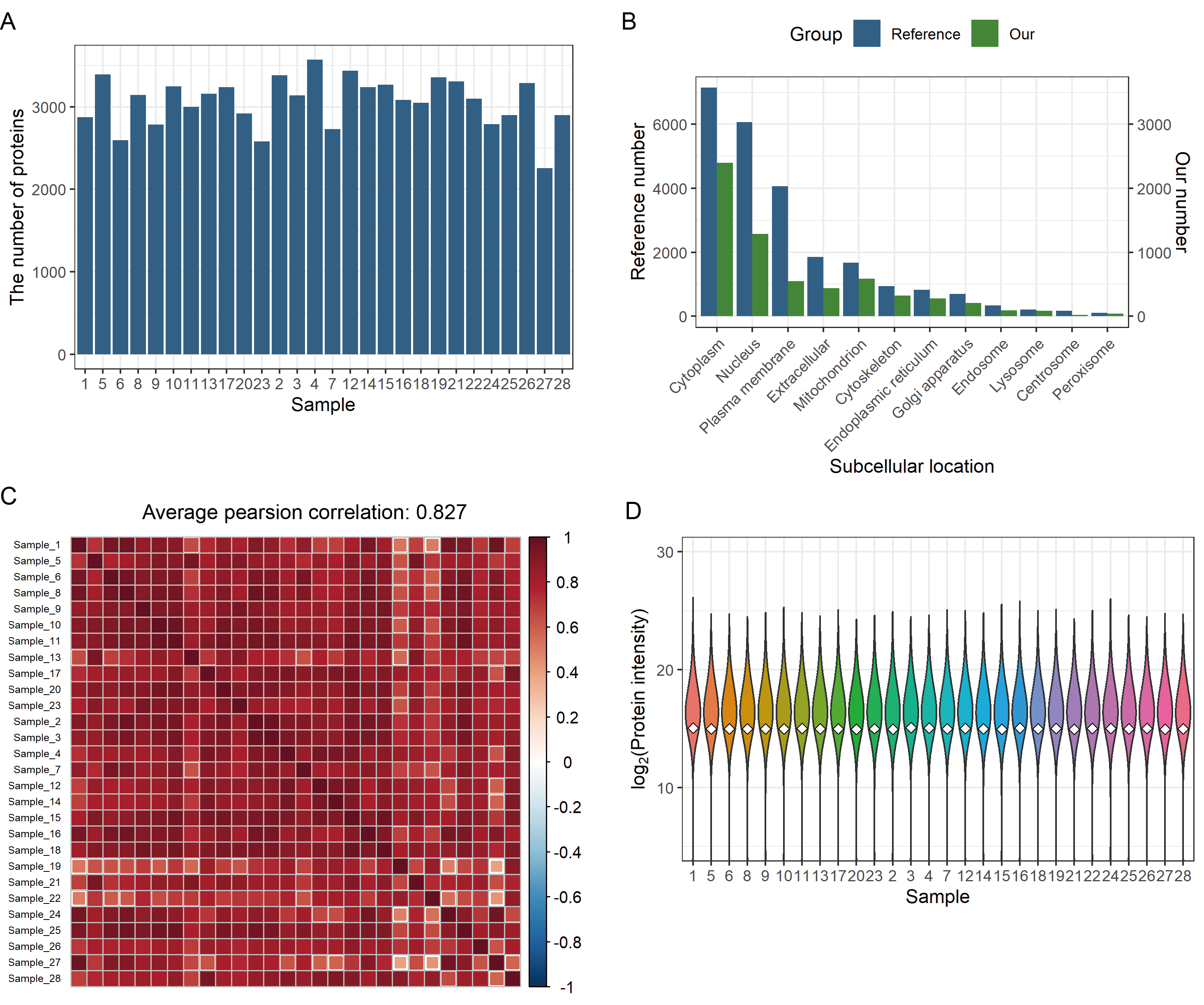


**Supplementary Figure 1.** Sample quality control. (A) Quantifiable amount of protein in each sample. (B) The distribution of the identified proteins in different subcellular organelles. (C) Pairwise correlation between samples. (D) The distribution of proteins intensity in each sample. The white diamond and the fill shape represent the intensity median and density distribution, respectively.


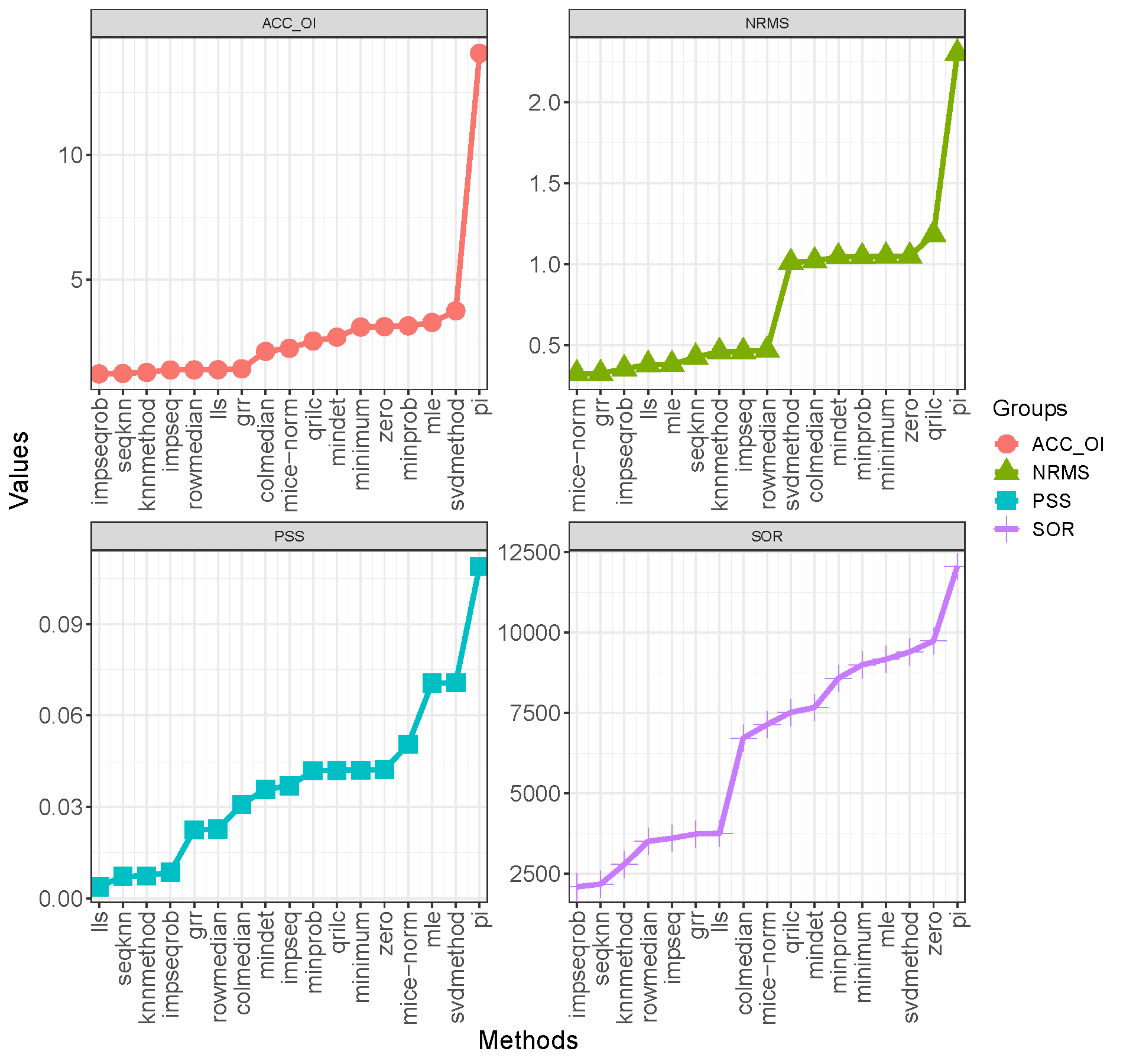


**Supplementary Figure 2.** Imputation performance of various methods evaluated by NAguideR.


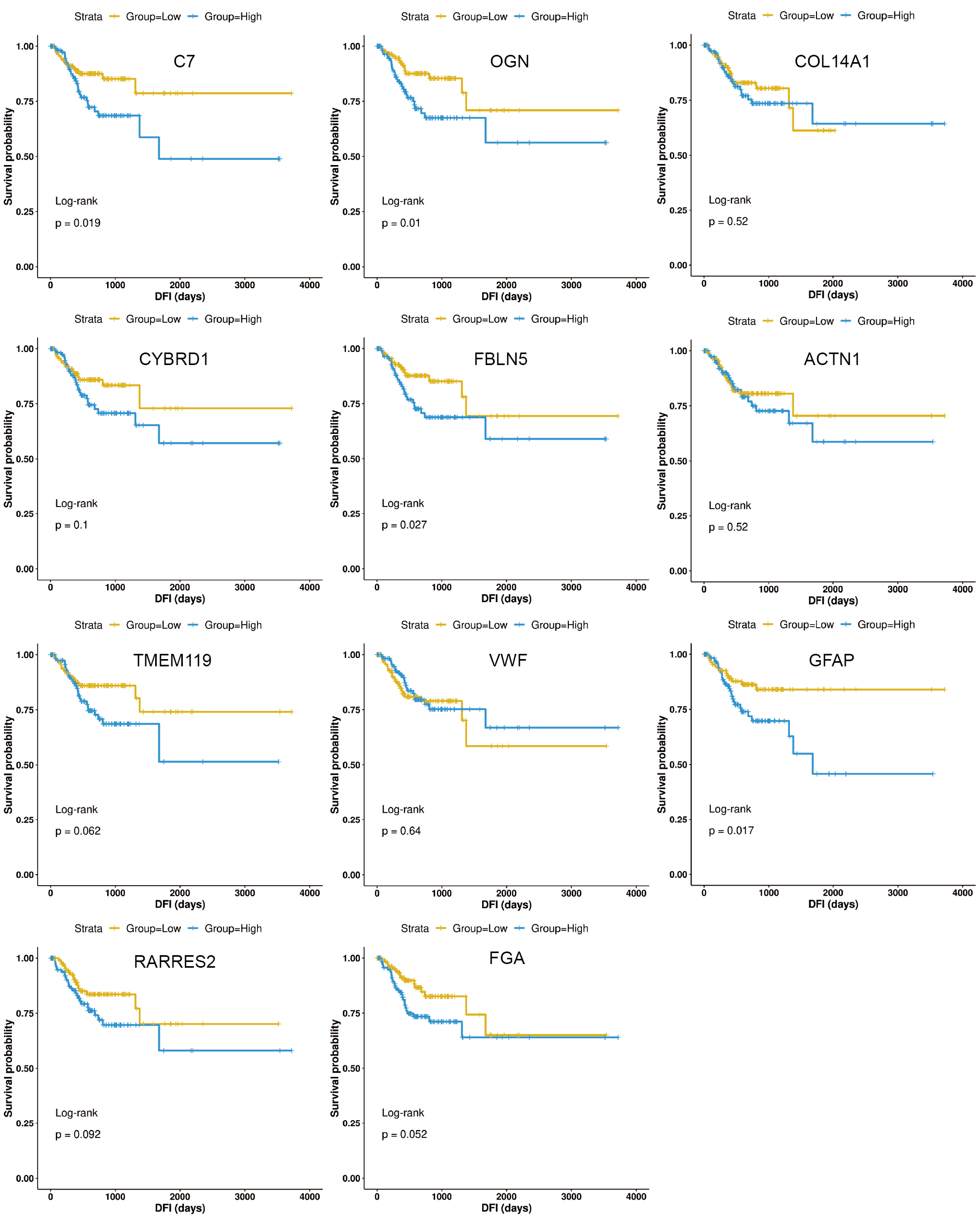


**Supplementary Figure 3.** Kaplan–Meier curves for the survival of GC patients with low and high gene expression. DFI, Disease-free interval.


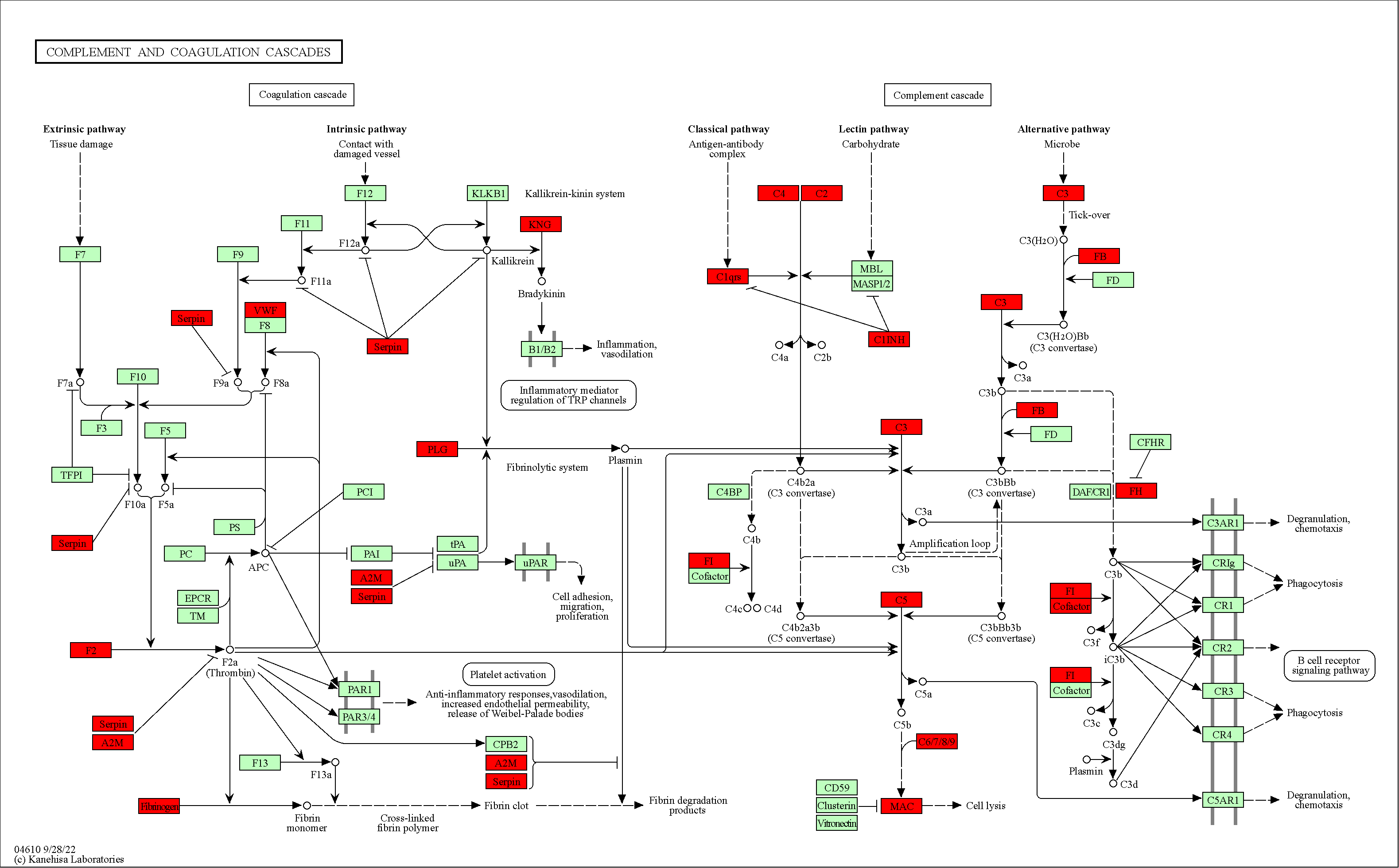


**Supplementary Figure 4.** “Complement and coagulation cascades” pathway form KEGG. The genes highlighted in red are represented in our data.


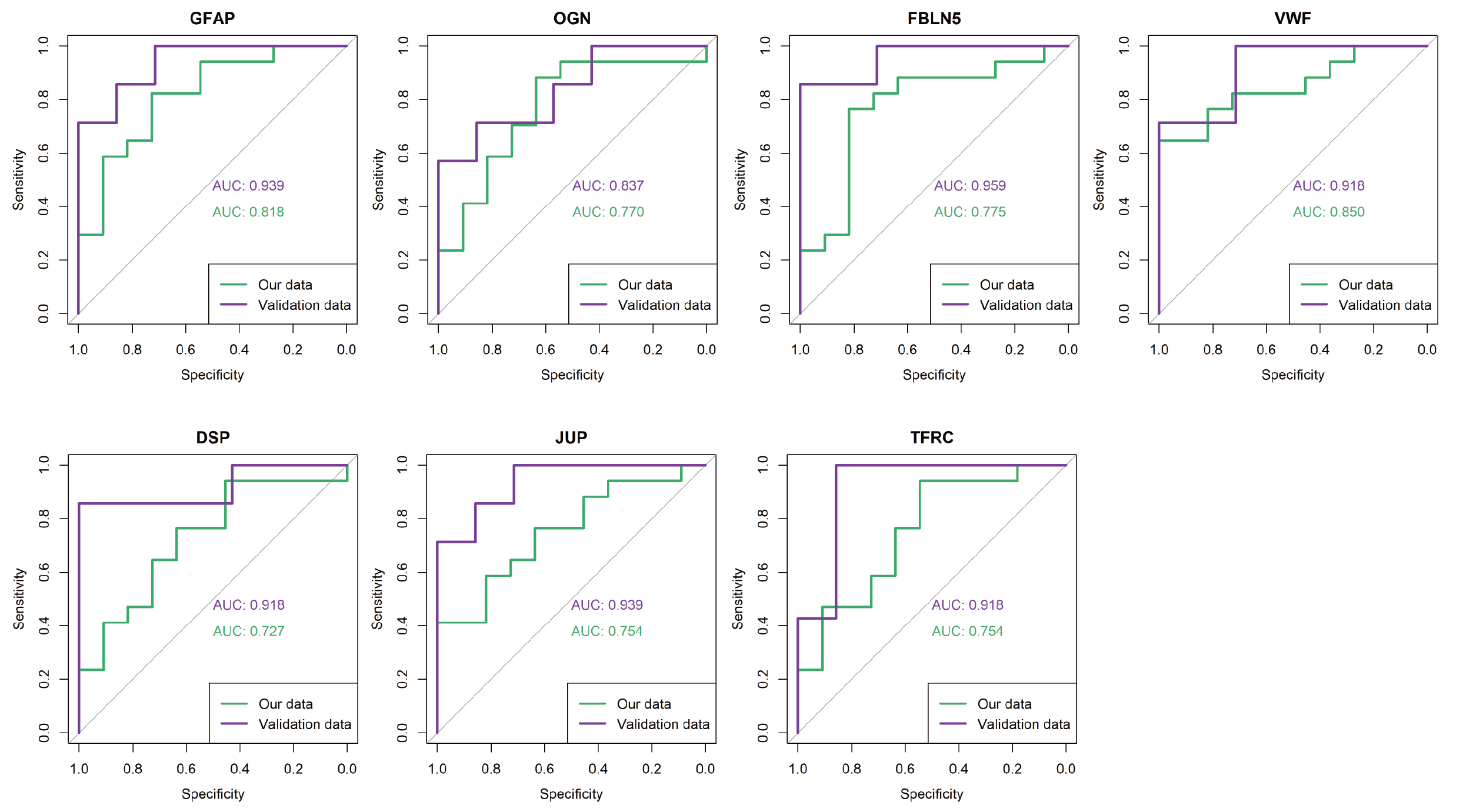


**Supplementary Figure 5.** ROC curves for the performance of the identified DEPs in our and validation cohort.


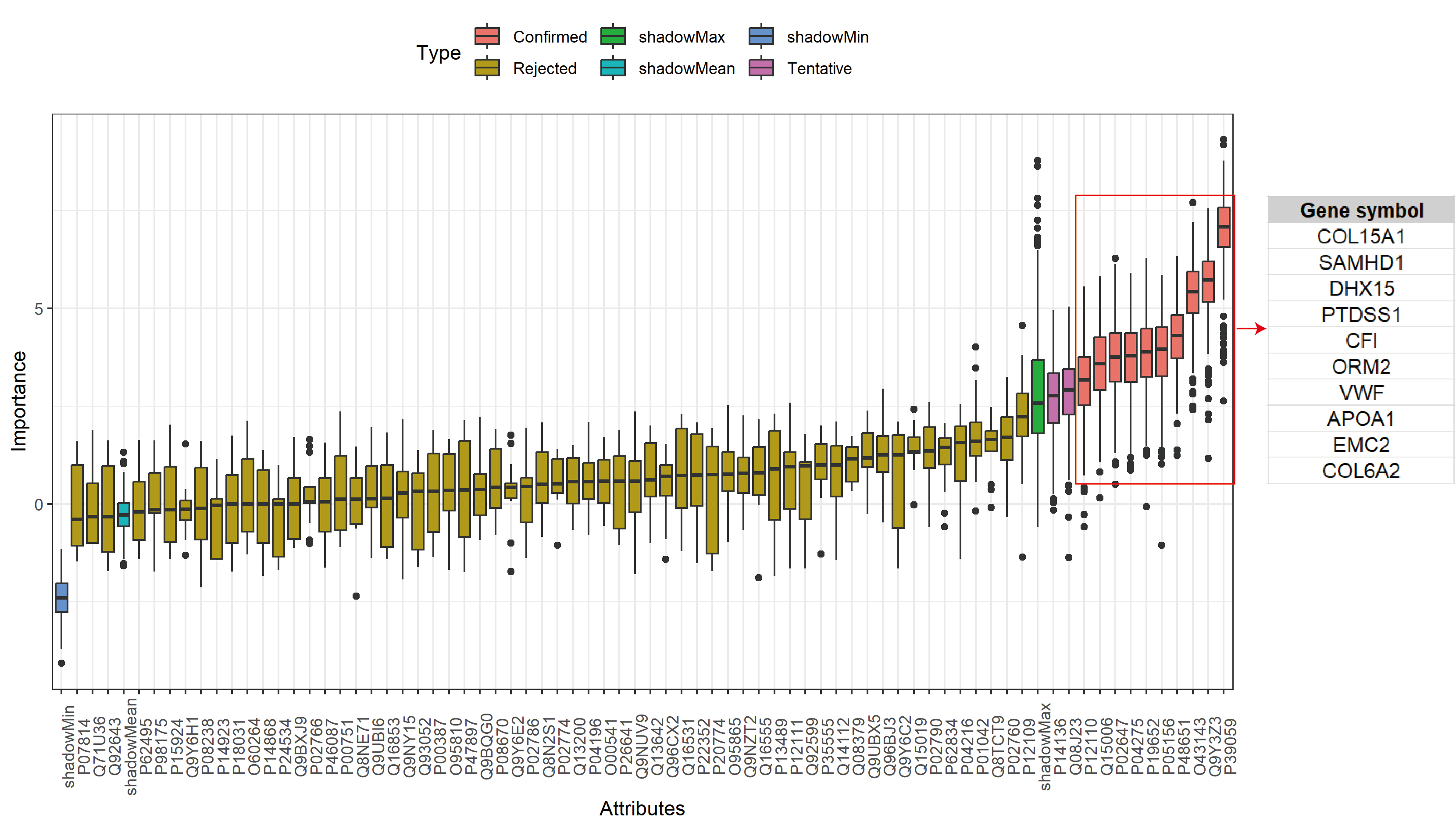


**Supplementary Figure 6.** The estimate of the importance of the variables used to predict the model.

**Supplementary method**

**Protein Extraction**

FFPE blocks were macro-dissected, deparaffinized with xylene, and washed with ethanol. The ethanol was removed completely, and the sections were left to air-dry. Following dewaxing, the samples were transferred into 1.5 ml centrifuge tubes and incubated with four volumes of lysis buffer (comprising 1% SDS and 1% protease inhibitors). Subsequently, sonication was performed to facilitate lysis. The resulting mixture was then centrifuged at 12,000 g for 10 minutes at 4°C to remove tissue debris, after which the supernatant was carefully transferred to new centrifuge tubes.

**Trypsin Digestion**

Equal amounts of protein from each sample were taken, and their volume was adjusted with lysis buffer until consistent. One volume of pre-cooled acetone was added and vortexed before an additional four volumes of pre-cooled acetone were introduced, then incubated at -20°C for two hours to allow precipitation. The mixture was centrifuged at 4500 g for five minutes and the supernatant discarded. The resulting precipitate was washed twice with pre-cooled acetone, allowed to dry, and resuspended in TEAB solution (final concentration 200 mM) using ultrasound to break up the pellet. Trypsin was added at a ratio of 1:50 (enzyme/protein, m/m), and the mixture was left overnight to allow digestion. Dithiothreitol (DTT) was added to reach a final concentration of 5 mM, followed by incubation at 56°C for thirty minutes to reduce disulfide bonds. Subsequently, iodoacetamide (IAA) was added to reach a final concentration of 11 mM, and the mixture was incubated at room temperature in the dark for fifteen minutes.

**LC-MS/MS Analysis**

Peptides were dissolved in mobile phase A and separated using the NanoElute UHPLC system. Mobile phase A was composed of 0.1% formic acid and 2% acetonitrile in water solution; mobile phase B contained 0.1% formic acid and 100% acetonitrile solution. The LC gradient was set as follows: 0-70 min, from 6% to 24% B; 70-84 min, from 24% to 35% B; 84-87 min, from 35% to 80% B; 87-90 min, maintained at 80% B, with flow rate kept at 450.00 nL/min. After separation by the UHPLC system, peptides were injected into a capillary ion source for ionization, followed by analysis on a tims-TOF Pro mass spectrometer. The ion source voltage was set at 2.0 kV, and both precursor ions and secondary fragments were detected and analyzed using high-resolution TOF. The scanning range of the MS/MS spectrum was set from 100 to 1700. Data acquisition mode used parallel accumulation serial fragmentation (PASEF) mode. For each primary MS scan, 10 PASEF scans were collected for precursor ions within charge states 0-5. To avoid repetitive scanning of precursor ions, dynamic exclusion time for MS/MS scanning was set at 30 s.

**Database Search**

The resulting raw data were processed using MaxQuant search engine. Search parameters were as follows: database Homo_sapiens_9606, with reverse decoy database enabled for calculating false discovery rate (FDR); common contaminants added to the database for removal of their influence in protein identification results. Trypsin/P enzyme specificity was chosen with up to two missed cleavages allowed; minimum peptide length was set to seven amino acids; maximum number of modifications per peptide was five; initial precursor ion mass tolerance was set to 20.0 ppm and main search tolerance to 20 ppm, while fragment ion mass tolerance was set to 20.0 ppm. Carbamidomethyl on Cys was specified as fixed modification and acetylation modification and oxidation on Met were specified as variable modifications. Protein quantitation method was set to LFQ and FDR threshold for protein was set to 1%.

**Survival Analysis**

Our R package “UCSCXenaShiny” was employed to plot the Kaplan–Meier survival curve with log-rank test using TCGA-STAD dataset [1]. The median of each gene expression was used to unbiasedly classify the patients into low and high groups.

**References**

[1] S. Wang, Y. Xiong, L. Zhao, K. Gu, Y. Li, F. Zhao, J. Li, M. Wang, H. Wang, Z. Tao, T. Wu, Y. Zheng, X. Li, X.-S. Liu, UCSCXenaShiny: an R/CRAN package for interactive analysis of UCSC Xena data, Bioinformatics. 38 (2021) 527–529. https://doi.org/10.1093/bioinformatics/btab561.
